# Dietary hydrolysable tannin improves intestinal health of largemouth bass (*Micropterus salmoides*): insights from NF-κB signaling pathway and arachidonic acid metabolism

**DOI:** 10.1186/s40104-025-01267-w

**Published:** 2025-10-23

**Authors:** Manqi Yang, Dahai Jiang, Zhangyi Xiao, Weibin Lai, Kai Chen, Shuwen Xu, Yuanyi Zuo, Liangliang Zhang, Liming Lu, Xiaoping Rao, Chunxiao Zhang, Jianchun Jiang

**Affiliations:** 1 College of Chemical Engineering, Huaqiao University, Xiamen, 361021 China; 2 Academy of Advanced Carbon Conversion Technology, Fujian Provincial Key Laboratory of Biomass Low-Carbon Conversion, Huaqiao University, No. 668 Jimei Avenue, Xiamen, 361021 China; 3 State Key Laboratory for Mariculture Breeding, Fisheries College of Jimei University, Xiamen, 361021 China; 4 Xiamen Key Laboratory for Feed Quality Testing and Safety Evaluation, Fisheries College, Jimei University, Xiamen, 361021 China; 5 Institute of Chemical Industry of Forest Products, CAF, Nanjing, 210042 China

**Keywords:** Arachidonic acid metabolism, Hydrolysable tannin, Intestinal inflammation, Largemouth bass, NF-κB signaling pathway, Plant-based protein source

## Abstract

**Background:**

To more effectively address the scarcity resources and elevated costs associated with fishmeal (FM), the utilization of cottonseed protein concentrate (CPC) as an alternative in aquaculture feeds has become increasingly prevalent. However, high levels of CPC substitution for FM have been reported to suppress the growth of fish and impair intestinal health. Hydrolysable tannin (HT) has been reported to exhibit biological activities such as anti-inflammatory and antioxidant activities, but whether the HT can generate positive biological effects on the intestinal health of largemouth bass (*Micropterus salmoides*) remains unknown. Largemouth bass (initial weight: 6.03 ± 0.01 g) were subjected to an 8-week feeding trial with three different diets: a basic diet (named as the NC), a high CPC diet (in which CPC replaced 75% of the FM protein in the NC diet, named as the HC), and an additive diet (1.25 g/kg of the HT was added to the HC diet, named as the HCH) to explore the potential benefits of HT on intestinal health.

**Results:**

The HC treatment significantly reduced the weight gain rate of fish, increased the feed conversion ratio, and induced intestinal inflammation. However, the HCH treatment could alleviate the adverse impacts of the HC diet, as evidenced by the promotion of growth and feed utilization, increased activity of digestive enzymes and antioxidant capacities, downregulated expression of pro-inflammatory factors, and maintenance of the integrity of intestinal barrier. Metabolomic analysis revealed that HCH treatment could reduce the pro-inflammatory active substances produced by arachidonic acid metabolism, including prostaglandin F_2α_ (PGF_2α_) and leukotriene B_4_ (LTB_4_). Transcriptomic results indicated that dietary HT might alleviate intestinal inflammation by suppressing the activation of the NF-κB signaling pathway. Furthermore, correlation analysis demonstrated that the metabolites PGF_2α_ and LTB_4_, derived from arachidonic acid, exhibited a significant positive correlation with the expression of genes associated with pro-inflammatory responses within the NF-κB signaling pathway.

**Conclusions:**

The study indicates that the HT mitigates the growth retardation and intestinal inflammation resulting from the HC diet on largemouth bass.

**Supplementary Information:**

The online version contains supplementary material available at 10.1186/s40104-025-01267-w.

## Introduction

The scarcity and high cost of fishmeal (FM), the primary protein source for aquatic animals, have led to a notable transition in aquaculture feeds towards the utilization of plant protein sources as substitutes for FM [1–4]. Cottonseed protein concentrate (CPC) is derived from the deep processing of cottonseeds [5]. It stands out among various plant protein sources due to its high protein content (over 60%), the presence of multiple vitamins, the relatively low content of the anti-nutritional factor gossypol, and its broad applicability, as evidenced by the application of CPC to various fish species with different feeding habits [6–8]. Specifically, CPC has been reported not to inhibit growth or exert adverse effects on physiological health of common carp (*Cyprinus carpio*) [6], rainbow trout (*Oncorhynchus mykiss*) [8], pearl gentian groupers (*Epinephelus fuscoguttatus* ♀ × *E. lanceolatus* ♂) [9], and largemouth bass [10] when used to replace 50% of the FM in diets. Nevertheless, it is important to note that there is a maximum threshold for the substitution of FM with CPC in aquaculture feed, as excessive levels of substitution lead to inhibited growth and intestinal injury in cultured fish [8, 10, 11]. Therefore, increasing the allowable proportion of CPC as a replacement for FM presents a significant challenge for the aquaculture feed industry.

For aquatic animals, the intestine plays a crucial role in maintaining health and enhancing productivity [12, 13]. It is distinguished by the presence of numerous villi that enhance the efficiency of nutrient digestion and absorption [14, 15]. Moreover, the intestine also serves as an immune organ to detect and defend against pathogens, maintain the integrity of intestinal barrier, regulate the immune responses, and promote the immune tolerance [12, 16]. The immune system can be bolstered by maintaining intestinal health, aided by the intestinal microbiota [17], which produces beneficial substances and suppresses the proliferation of pathogenic bacteria [16]. In addition, promoting intestinal health in aquatic animals can reduce the reliance on pharmaceuticals and antibiotics in aquaculture practices [15, 18, 19]. Therefore, the preservation of intestinal health in aquatic animals is a vital approach for enhancing the efficiency and sustainability of aquaculture industry.

Plant extracts have been commonly utilized as feed additives in the aquaculture to enhance the survival rate and improve intestinal health of aquatic animals [20–22]. Hydrolysable tannin (HT), a polyphenolic compound extracted from plants, is utilized as a feed additive due to its antioxidative, anti-inflammatory, and immune-enhancing attributes [20]. There was evidence that the dietary HT enhanced the non-specific immune response and intestinal wall thickness in whiteleg shrimp (*Penaeus vannamei*) [23], promoted the secretion of intestinal trypsin and α-amylase by upregulating the mRNA expression of intestinal target of rapamycin (*tor*) in grass carp (*Ctenopharyngodon idellus*) [24], and enhanced the activity of intestinal superoxide dismutase and upregulated the gene expression levels of intestinal anti-inflammatory factor in grass carp [25]. Furthermore, the dietary HT has been reported to mitigate the elevated mRNA expression levels of intestinal tumor necrosis factor-α (*tnf-α*) and cyclooxygenase 2 (*cox2*) induced by K-carrageenan in zebrafish, and attenuated intestinal inflammation by suppressing the activation of the NF-κB pathway [18]. The COX2 is a metabolite derived from arachidonic acid, facilitates the production of various pro-inflammatory metabolites [26, 27], which activates the NF-κB signaling pathway. Upon activation, the NF-κB signaling pathway can bind to the promoter region of COX2, enhancing its transcription and consequently increasing the synthesis of pro-inflammatory products within arachidonic acid metabolism, thereby intensifying the inflammatory response [28, 29]. Therefore, the addition of appropriate amounts of HT in diets has the potential to promote the intestinal health in aquatic animals.

*Micropterus salmoides*, commonly referred to as the largemouth bass, is a rapidly growing and palatable freshwater carnivorous fish [30]. Since its introduction from North America, it has been extensively cultivated in central and eastern China owing to its high adaptability, as evidenced by its production has surpassed 888,000 tons by 2023, thereby emerging as a significant economic fish species [31]. Condensed tannins, which have a relatively large molecular weight and degree of polymerization and thus possesses a strong ability to bind with proteins, are regarded as an antinutritional factor [20]. However, existing research has found that adding 3.75 g/kg of condensed tannin to the diet could alleviate intestinal inflammation in largemouth bass [31]. Nevertheless, the chemical structure of the HT presents certain advantages compared to the condensed tannin, and differences in chemical structure may also lead to variations in metabolic products [20]. Consequently, it remains unclear whether the HT can also promote the intestinal health of largemouth bass. Therefore, this research is designed to examine the potential of the dietary HT to alleviate the adverse impacts of the high CPC diet on the growth performance and intestinal health of juvenile largemouth bass. It aims to contribute to the body of knowledge regarding the increased substitution of FM with CPC in aquaculture feeds, thereby enhancing the economic viability and alleviating the pressure on global fisheries resources.

## Materials and methods

### HT and condensed tannin preparation

The methods for the extraction and purification of the HT and condensed tannin were detailed in our earlier studies [32, 33]. The purity of the HT extracted from gallnuts and condensed tannin extracted from the bark of quebracho was ultimately determined to be 90.25% and 70%, respectively.

### Comparative analysis of bioactive constituents between HT and condensed tannin

Following dissolution in methanol solution, the samples were centrifuged to obtain the supernatant, which was analyzed by LC-MS (Exion LC/QTRAP 6500+, SCIEX, USA). The chromatographic parameters were established as follows: an Xselect HSS T3 column (dimensions: 2.1 mm × 150 mm, particle size: 2.5 μm) was employed. The mobile phases consisted of 0.1% formic acid in water (mobile phase A) and 0.1% formic acid in acetonitrile (mobile phase B). The gradient elution was programmed as follows: 98% A and 2% B from 0 to 2 min; transitioning to 0% A and 100% B from 15 to 17 min; and reverting to 98% A and 2% B from 17.1 to 20 min. The column temperature was maintained at 50 °C, with a flow rate of 0.4 mL/min. The mass spectrometry parameters were configured as follows: in the positive ion mode, the curtain gas was set to 35 psi, the collision gas was adjusted to medium, the ion spray voltage was maintained at 5,500 V, the temperature was controlled at 550 °C, and both ion source gas 1 and ion source gas 2 were set to 60. For the negative ion mode, the ion spray voltage was modified to −4,500 V, while all other conditions remained consistent with those employed in the positive ion mode. Subsequently, the integration and calibration of the chromatographic peaks were carried out (GeneDenovo Biotechnology Co., Ltd., China).

### Preparation of diets and management of feeding trial

The three experimental diets are as follows: a basic diet (named the NC), a high CPC diet (CPC substituted 75% of the FM protein in the NC diet, named the HC), and an additive diet (adding 1.25 g/kg of the HT to the HC diet, named the HCH). The levels of CPC replacing FM and the dosage of HT supplements were based on previous experiments [31, 33]. The composition of experimental diets is showed in Table 1. The non-oil diet ingredients were meticulously blended in sequential stages, subsequently amalgamated with 25% water, and extruded into floating pellets utilizing a twin-screw extruder (TSE65, Yanggong Machinery, Beijing, China). The pellets underwent a drying process at 60 °C, followed by the application of fish oil via spraying.

**Table 1:** Formulation and proximate composition of experimental diets (dry matter basis)

Item	NC	HC	HCH
Ingredients, g/kg			
Fish meal	430.00	107.50	107.50
Corn starch	90.00	90.00	90.00
Soybean meal	200.00	200.00	200.00
Casein	90.00	90.00	90.00
Cottonseed protein concentrate	0.00	360.10	360.10
HT	0.00	0.00	1.25
Ca(H_2_PO_4_)_2_	10.00	10.00	10.00
Vitamin mixture^1^	4.00	4.00	4.00
Mineral premix^2^	6.00	6.00	6.00
L-Ascorbate-2-phosphate	0.20	0.20	0.20
Microcrystalline cellulose	106.3	41.50	40.25
Choline chloride	5.00	5.00	5.00
L-Lysine HCl	0.00	6.40	6.40
DL-Methionine	0.00	2.80	2.80
Y_2_O_3_	1.00	1.00	1.00
Antiseptic	0.50	0.50	0.50
Fish oil	57.00	75.00	75.00
Proximate compositions, %			
Crude protein	46.03	46.11	46.55
Crude lipid	10.41	10.35	10.39
Total tannin, mg/kg	500.51	510.26	1628.82

^1^Vitamin premix (mg/kg): thiamin, 10 mg; riboflavin, 8 mg; pyridoxine HCl, 10 mg; vitamin B_12_, 0.2 mg; vitamin K_3_, 10 mg; inositol, 100 mg; pantothenic acid, 20 mg; niacin acid, 50 mg; folic acid, 2 mg; biotin, 2 mg; retinol acetate, 400 mg; cholecalciferol, 5 mg; alpha-tocopherol, 100 mg; wheat middling, 132.8 mg

^2^Mineral premix (mg/kg): KCl, 200 mg; KI, 60 mg; CoSO_4_, 100 mg; CuSO_4_·5H_2_O, 24 mg; FeSO_4_·H_2_O, 400 mg; ZnSO_4_·H_2_O, 174 mg; MnSO_4_·H_2_O, 78 mg; MgSO_4_·7H_2_O, 800 mg; Na_2_SeO_3_, 50 mg; zoelite, 3,114 mg

The experimental largemouth bass were procured from a hatchery (Zhangzhou, China). The aquaculture facilities utilized for this study were provided by Xiamen Jiakang Feed Co., Ltd. (China). The fish were initially reared on the NC diet in aquaria for a duration of two weeks to acclimate them to the rearing environment. Following this acclimation period, healthy fish (initial weight: 6.03 ± 0.01 g) were allocated into the aquaria (25 fish per aquarium, with 100 L freshwater; each diet treatment group × three aquaria). The fish were administered the experimental diet over a period of eight weeks. During the feeding experiment, the fish were fed twice daily (8:30 and 17:30) until apparent satiation. The water quality parameters in the rearing aquaria were consistently maintained, with water temperature of 24 ± 2 °C, pH of 7.0 ± 0.3, and dissolved oxygen levels at 6.8 ± 0.2 mg/L.

### Sample collection

After the 8-week of feeding, the fish was fasted for a 24-h prior to anesthesia (0.01% eugenol). Subsequently, 10 fish were randomly selected from each aquarium for the measurement of body length and weight. Blood samples were then collected, and the handling of blood samples adhered to protocols established in the previous study [7]. The mid-intestines of 10 fish per aquarium were promptly frozen. Of these, 3 mid-intestines were allocated for biochemical analysis, 3 for gene expression level analysis, and the remaining 4 for intestinal microbiota, metabolite analysis, and transcriptome analysis. Additionally, the mid-intestines from 3 additional fish per aquarium were fixed in 4% paraformaldehyde for examination of intestinal structural integrity. To ascertain the basic nutritional composition of the fish, 3 fish from each aquarium were frozen at −20 °C.

### Growth performance and whole-body component analysis

Calculating the indicators related to growth for the fish in each experimental group. The formulas for these calculations are as follows:

$$\begin{array}{c}Weight\;gain\;rate\;\left(WGR,\%\right)=\left(FBW\;-\;IBW\right)/IBW\times100;\\Survival\;rate\;\left(SR,\%\right)=FFN/IFN\times100;\\Feed\;conversion\;ratio\;\left(FCR\right)=feed\;intake/\left(FBW-IBW\right);\\Feed\;intake\;\left(FI,g/fish\right)=feed\;intake/fish\;number;\\Condition\;factor\;\left(CF,g/cm^3\right)=FBW/FBL^3\times100.\end{array}$$

In which, FBW: final body weight; IBW: initial body weight; FFN: final fish number; IFN: initial fish number; FBL: final body length.

The examination of the proximate components of the three diets and fish was conducted in accordance with AOAC guidelines [34]. Specifically, moisture content was determined by drying at 105 °C to constant weight. The Kjeldahl method was used to measure the crude protein content. A mixed indicator comprising methyl red and bromocresol green was employed, with hydrochloric acid serving as the titrant. Post sulfuric acid digestion, an automated Kjeldahl nitrogen analyzer (K1100, Hanon, China) was utilized to analyze the samples. Soxhlet extraction was used to measure the crude lipid content. Anhydrous ether was added to the Soxhlet extractor containing the sample, ensuring the samples were fully submerged. Subsequently, the extractor was positioned within a water bath. A constant temperature of 50 °C was maintained, and continuous extraction was carried out for 6 h. Ash content was measured by muffle furnace calcination method. The carbonized sample undergoes calcination in a muffle furnace (MFLC-4/16D, Taisite, China) at a temperature of 550 °C for a duration of 4 h. Upon removal from the furnace, the sample was transferred to a desiccator. When it had cooled to ambient temperature, the sample was subsequently weighed.

### Biochemical analysis

The intestinal tissues used for biochemical analysis were all homogenized according to the instruction of the kit used for each indicator. Intestinal lipase (A054-2-1), α-amylase (C016-1-2), maltase (A082-3-1), total glutathione (T-GSH; A061-1-2), total antioxidant capacity (T-AOC; A015-2-1), total superoxide dismutase (T-SOD; A001-1-2), and malondialdehyde (MDA; A003-1-2) levels and serum D-lactic acid (A019-3-1) content, were determined using commercial kits (Nanjing Jiancheng Bioengineering Institute, China). Serum diamine oxidase (DAO; JL45614), lysozyme (LZM; JL54355), and lipopolysaccharide (LPS; JL13861) levels, as well as the content of intestinal trypsin (JL22682) and leucine aminopeptidase (JL22631), were quantified by ELISA kits (Jianglai Biology, Shanghai, China). Intestinal prostaglandin A_2_ (PGA_2_; BP04871), prostaglandin F_2α_ (PGF_2α_; BP04885), leukotriene B₄ (LTB_4_, BP04860); COX2 (BP04889), and lipoxygenases (LOX, BP04884) were quantified by ELISA kits (Shanghai Baipeng Biotechnology Co., Ltd., China). The experimental operations were precisely adhered to protocols outlined in the reagent kit instructions, and a multimode microplate reader (Infinite E Plex, Tecan, Switzerland) was used for detection.

### Histological analysis

Intestinal tissues fixed in 4% paraformaldehyde were used to prepare hematoxylin and eosin (H&E) stained sections as well as TUNEL fluorescent stained sections, following methods consistent with previous studies [31]. Briefly, the intestinal tissues underwent dehydration via a graded series of alcohol concentrations, followed by transparency with xylene, and subsequent embedding in paraffin. After sectioning (6 μm thick), the sections were stained with hematoxylin and eosin. For TUNEL fluorescent stained sections, following the dewaxing of the paraffin sections, sections were incubated with a proteinase K for 20 min at 37 °C. Subsequently, the sections were rinsed with PBS. After equilibration to room temperature, a TUNEL kit (G1502, Servicebio, China) was employed for the labeling reaction. Post-labeling, the sections underwent PBS washing, after which DAPI staining solution (G1012, Servicebio, China) was applied, and the sections were incubated in the dark at room temperature for 10 min. The sections were then washed with PBS, air-dried, and mounted. Finally, the sections were examined and analyzed using a microscope (DMIL LED, Leica, Germany). Additionally, the Leica image processing software was employed to quantitatively assess pertinent metrics of H&E-stained sections, including the number, length, and width of intestinal villi, as well as the thickness of the intestinal muscularis and lamina propria.

### Gene expression analysis

Total RNA was extracted from intestinal tissues utilizing an RNA extraction kit (AG 21017, Accurate Biotechnology (Hunan) Co., Ltd., Changsha, China). Following quality assessment, the RNA was reverse-transcribed into cDNA. qPCR (Bio-Rad CFX96, USA) was performed to detect the target genes using the PreMix qPCR Kit (AG 11718, Accurate Biotechnology (Hunan) Co., Ltd., Changsha, China). The qPCR protocol is outlined as follows: initially, a pre-denaturation step was conducted at 95 °C for 30 s. This was followed by 40 cycles, each comprising a denaturation phase at 95 °C for 10 s, and an annealing and extension phase at 60 °C for 30 s. Primer design was based on previous studies conducted in largemouth bass (Table S1).

### Intestinal microbiota analysis

The analysis method was consistent with that of the previous study [31]. After extracting DNA from intestinal tissues, the resultant PCR products were subsequently purified, quantified, and sequenced to facilitate Illumina library preparation (Genedenovo Biotechnology Co., Ltd., China).

### Intestinal metabolites analysis

Intestinal samples were mixed with methanol solution, followed by cryogenic grinding and ultrasonic extraction. After centrifugation, the supernatant was collected and subjected to nitrogen blow-drying. The processed samples, after undergoing oximation and derivatization reactions, were analyzed by LC-MS (UHPLC-Orbitrap Exploris 240, Thermo Fisher, USA). Data were processed before identified metabolite with databases, including HMDB (http://www.hmdb.ca/), KEGG (https://www.kegg.jp/kegg/pathway.html) and Metlin (https://metlin.scripps.edu/) (Mejorbio Co., Ltd., China).

### Intestinal transcriptome analysis

Total RNA was extracted from intestinal tissues using Trizol. After mRNA enrichment, fragmentation was carried out. Subsequently, cDNA was synthesized by reverse transcription, and adapters were ligated. After sorting, PCR amplification was performed. The purified products obtained were used for sequencing and bioinformatics analysis (Mejorbio Co., Ltd., China).

### Correlation analysis

To elucidate gene-metabolite interaction networks, a Pearson correlation analysis was conducted between differentially expressed genes obtained from the transcriptome and metabolites. The Mantel test was used to analyze the associations among the indicator species of microorganisms at the species level, differential genes, and metabolites.

### Statistical analysis

An one-way analysis of variance (ANOVA), followed by a Tukey post hoc test, was used to assess the data (SPSS 20.0). Significant differences between the datasets were identified using a threshold of *P* < 0.05. The data are presented as means ± standard deviation (SD).

## Results

### Analysis of metabolites of HT and condensed tannin

In both categories of tannins, the principal metabolites include organic acids and its derivatives, amino acids and its derivatives, flavonoids, carbohydrates and its derivatives, lipids, organoheterocyclic compounds, phenolic acids, alkaloids and its derivatives, vitamins, phenols and its derivatives, as well as nucleotides and its derivatives (Fig. S1A). Notably, the abundances of metabolites such as organic acids and its derivatives, carbohydrates and its derivatives, organoheterocyclic compounds, and vitamins were significantly elevated in HT compared to condensed tannin (Fig. S1A; *P* < 0.05). Conversely, the metabolites of flavonoids, lipids, and phenols and its derivatives were more prevalent in condensed tannin (Fig. S1A; *P* < 0.05). Furthermore, variable importance in projection (VIP) analysis identified gallic acid as the metabolite exhibiting the most significant differential presence between the two types of tannins (Fig. S1B; *P* < 0.05).

### Growth performance and whole-body composition

Fish in the HC group demonstrated a significantly lower weight gain rate compared to those in the NC and HCH groups, whereas the feed conversion ratio exhibited an opposite trend (Table 2; *P* < 0.05). The feed intake of the HC group was not notably different from that of the HCH group (Table 2; *P* > 0.05), but both were lower than that of the NC group (Table 2; *P* < 0.05). The condition factor and survival rate of fish exhibited no marked variations among the different experimental groups (Table 2; *P* > 0.05).

As shown in Table 2, dietary treatments did not markedly affect the moisture, crude lipid, and ash in the whole-body composition of fish (*P* > 0.05). In comparison to fish in the NC and HCH groups, fish in the HC group exhibited reduced crude protein levels in the whole-body composition (*P* < 0.05).

**Table 2:** Growth performance and whole-body composition of largemouth bass

Items	Diets		
	NC	HC	HCH
Growth performance			
Survival rate, %	100.00 ± 0.00	100.00 ± 0.00	100.00 ± 0.00
WGR, %	579.94 ± 27.47^a^	475.25 ± 15.99^c^	525.01 ± 6.01^b^
FI, g/fish	36.54 ± 1.41^a^	30.68 ± 1.05^b^	32.68 ± 0.25^b^
FCR	1.04 ± 0.01^b^	1.07 ± 0.01^a^	1.04 ± 0.01^b^
CF, g/cm^3^	1.51 ± 0.04	1.48 ± 0.02	1.61 ± 0.08
Whole-body, % wet weight			
Moisture	73.87 ± 3.22	73.07 ± 2.12	73.66 ± 1.84
Crude protein	17.23 ± 0.05^a^	15.48 ± 0.44^b^	16.89 ± 0.21^a^
Crude lipid	7.66 ± 0.52	7.40 ± 0.41	7.06 ± 0.14
Ash	4.48 ± 0.37	4.81 ± 0.42	4.55 ± 0.41

*WGR* Weight gain rate, *FI* Feed intake, *FCR* Feed conversion ratio, *CF* Condition factor. Data in same row with different letters indicates significant differences (*P* < 0.05), *n* = 3. NC: the basic diet. HC: the high CPC diet (CPC substitutes 75% of the FM protein in the NC diet). HCH: addition of 1.25 g/kg of HT to the HC diet

### Intestinal integrity

**Fig. 1:**
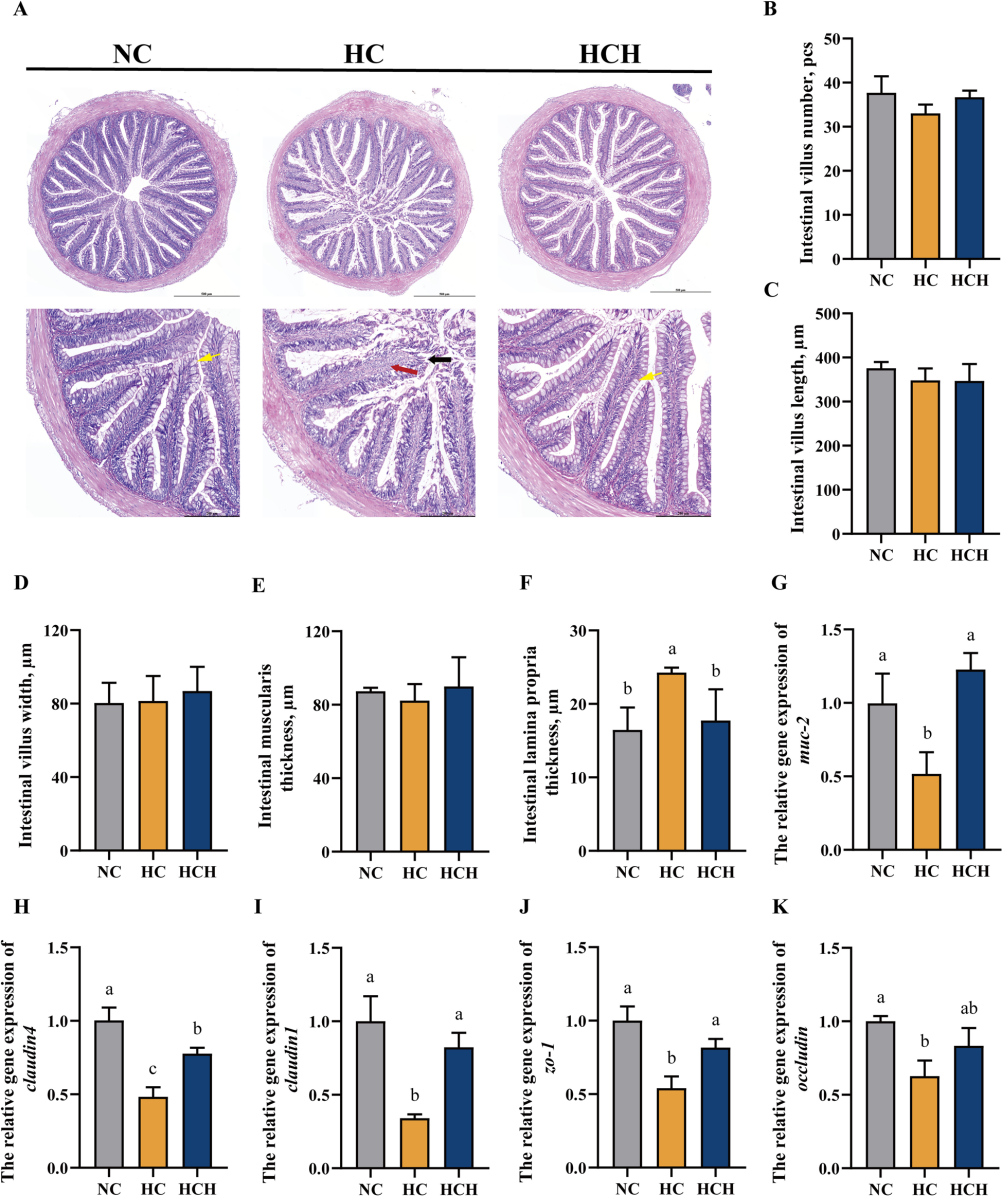
Analysis of the integrity of the intestinal structure. **A** Intestinal histological structure (H&E, scale bars: 500 and 200 μm), the black arrow point to damaged area, the red arrow point to lamina propria, the yellow arrow point to the goblet cells. **B** Intestinal villus number. **C** The length of intestinal villus. **D** The width of intestinal villus. **E** The thickness of muscularis in intestine. **F** The thickness of lamina propria in intestinal villus. **G** The relative gene expression of mucin-2 (*muc-2*). **H** The relative gene expression of *claudin4*. **I** The relative gene expression of *claudin1*. **J** The relative gene expression of zonula occludens-1 (*zo-1*). **K** The relative gene expression of *occludin*. Values are mean ± SD, *n* = 3. The significant differences among groups are represented by different lowercase letters on the column (*P* < 0.05). NC: the basic diet; HC: CPC substitutes 75% of the FM protein in the NC diet; HCH: adding 1.25 g/kg of HT to the HC diet

Under the HC diet treatment, the villi in the fish intestine were damaged, while the intestinal structure of fish under the NC and HCH diet treatments remained intact (Fig. 1A). In comparison to the NC and HCH diet treatments, the HC diet treatment did not significantly change the number, length, and width of villi, as well as the thickness of muscularis in the intestine of fish (Fig. 1B–E; *P* > 0.05). However, fish under the HC diet had the thickest intestinal lamina propria than those under the NC and HCH diet treatments (Fig. 1F; *P* < 0.05). Moreover, the gene expression of the mucin-2 (*muc-2*) was significantly downregulated in the HC group compared with the NC and HCH groups (Fig. 1G; *P* < 0.05). Comparatively with fish under the NC and HCH diet treatments, those on the HC diet had significantly lower expression levels of tight junction protein genes (*claudin4*, *claudin1*, and zonula occludens-1 (*zo-1*)) in intestine (Fig. 1H–J; *P* < 0.05). However, in comparison to the HC diet, the HCH treatment did not improve the relative expression levels of *occludin* (Fig. 1K; *P* > 0.05).

**Fig. 2:**
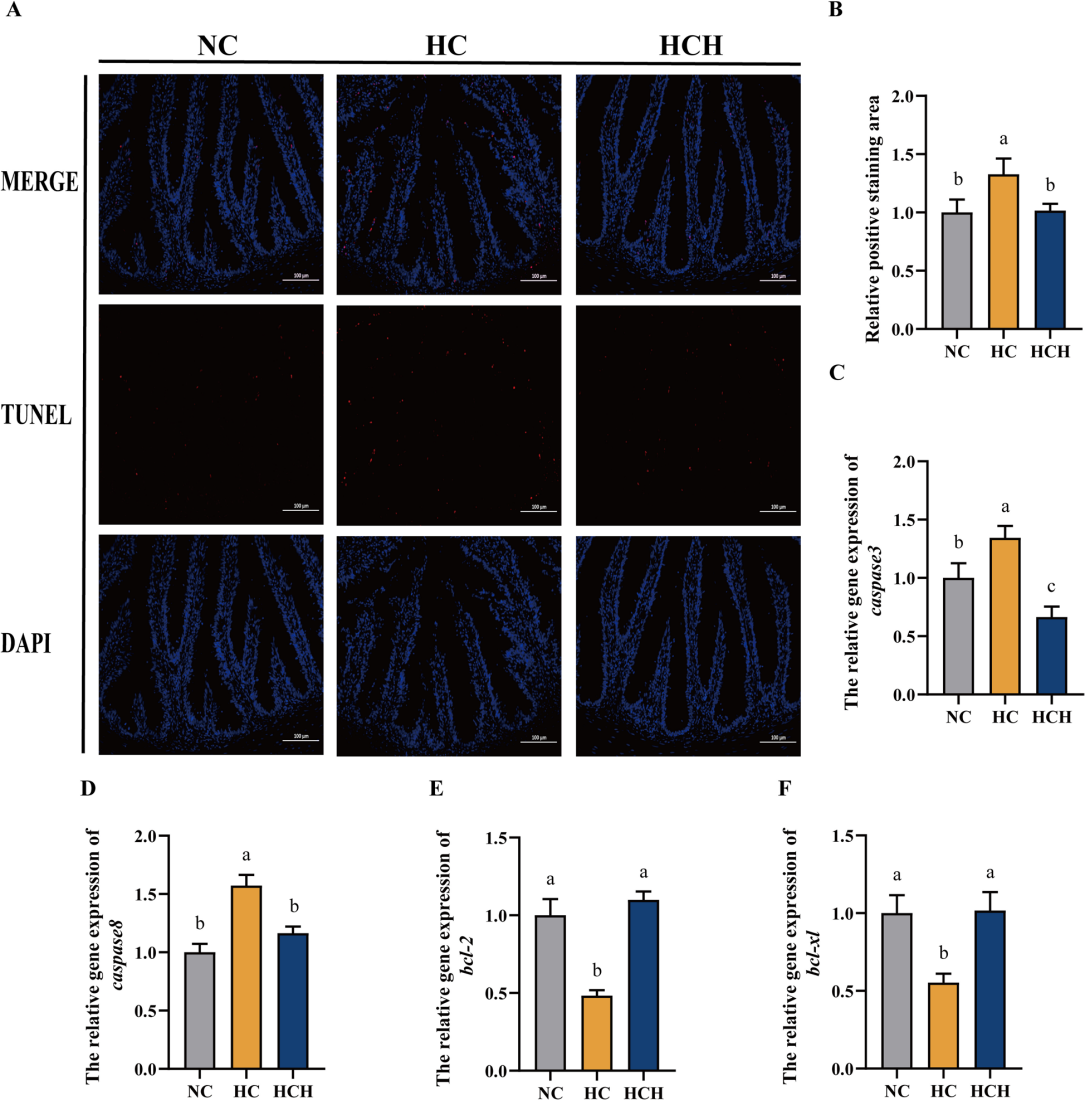
The apoptosis in the intestines of largemouth bass. **A** Intestinal histological structure by TUNEL (scale bars: 100 μm), with red highlight means TUNLE-positive cells. **B** Relative positive staining area. **C** The relative gene expression of *caspase3*. **D** The relative gene expression *caspase8*. **E** The relative gene expression of B-cell lymphoma-2 (*bcl-2*). **F** The relative gene expression of B-cell lymphoma-extra large (*bcl-xl*). Values are mean ± SD, *n* = 3. The significant differences among groups are represented by different lowercase letters on the column (*P* < 0.05). NC: the basic diet; HC: CPC substitutes 75% of the FM protein in the NC diet; HCH: adding 1.25 g/kg of HT to the HC diet

According to the TUNEL staining results, the intestinal staining-positive area of fish treated with HC diet was significantly higher than that in the other two groups (Fig. 2A and B; *P* < 0.05). The expression of pro-apoptotic genes (*caspase3* and *caspase8*) in the intestine of fish treated with HC diet was significantly upregulated than that in fish treated with NC diet and HCH diet, whereas the expression of anti-apoptotic genes [B-cell lymphoma-2 (*bcl-2*) and B-cell lymphoma-extra large (*bcl-xl*] showed the opposite trend (Fig. 2C–F; *P* < 0.05).

### Intestinal digestive enzymes

**Fig. 3:**
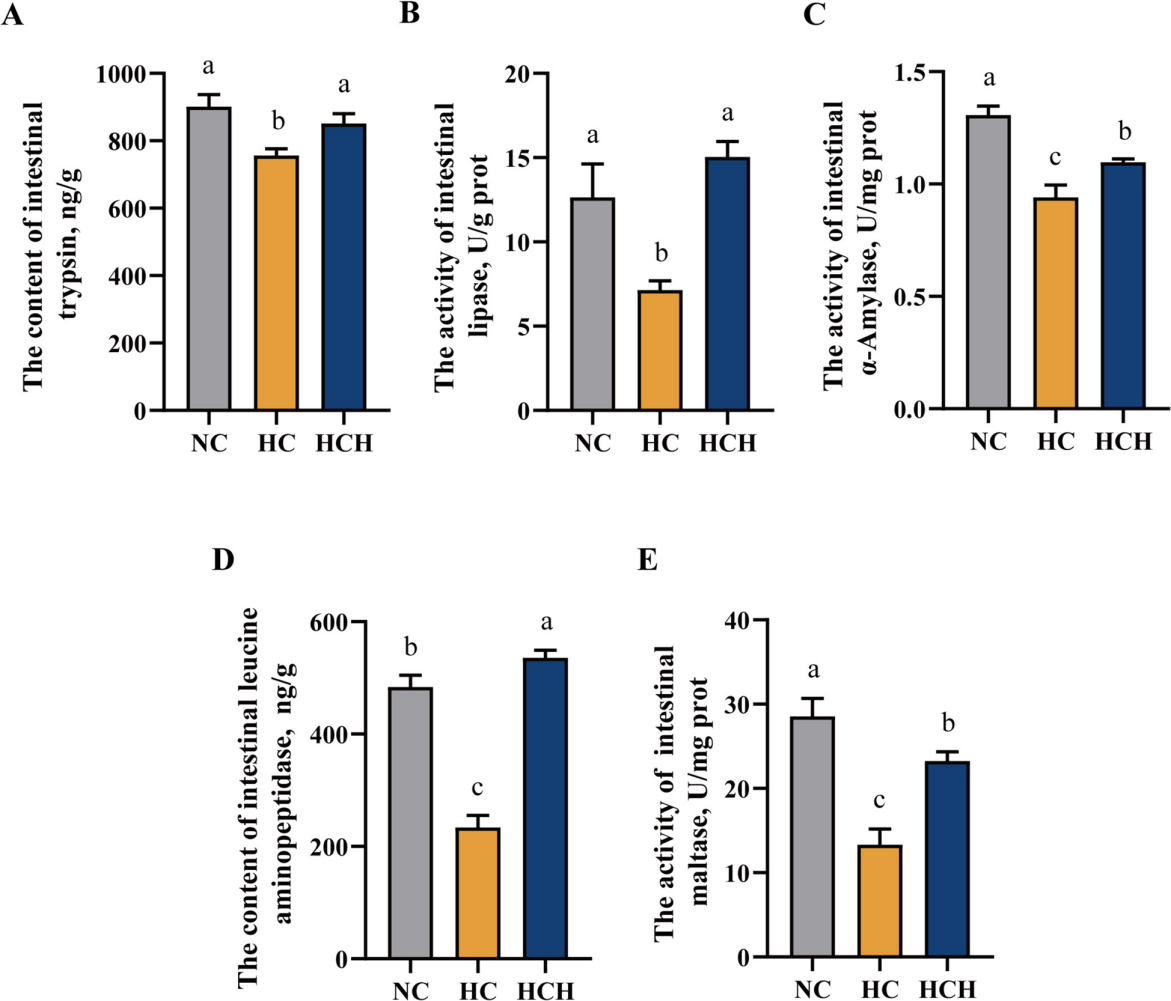
Effects of HT supplementation in the high CPC diet on intestinal digestive enzymes. **A** The content of trypsin, ng/g. **B** The activity of lipase, U/g prot. **C** The activity of α-amylase, U/mg prot. **D** The content of leucine aminopeptidase, ng/g. **E** The activity of maltase, U/mg prot. Values are mean ± SD, *n* = 3. The significant differences among groups are represented by different lowercase letters on the column (*P* < 0.05). NC: the basic diet; HC: CPC substitutes 75% of the FM protein in the NC diet; HCH: adding 1.25 g/kg of HT to the HC diet

In the HC group, fish were found to have decreased levels of trypsin and leucine aminopeptidase in the intestine, as well as lower activities of lipase, α-amylase, and maltase compared with fish in the NC and HCH groups (Fig. 3A–E; *P* < 0.05).

### Intestinal antioxidant defense system

**Fig. 4:**
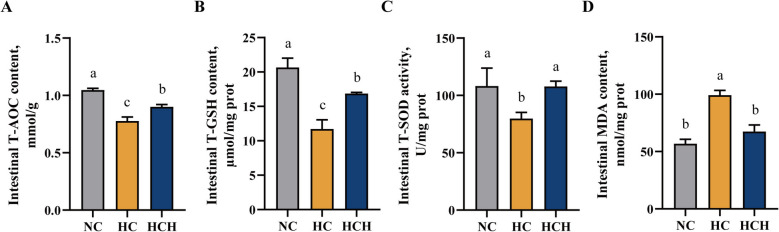
Effect of HT supplementation in the high CPC diet on the antioxidant capacity of the intestine. **A** The content of total antioxidant capacity (T-AOC, mmol/g). **B** The content of total glutathione (T-GSH, μmol/mg prot). **C** The activity of total superoxide dismutase (T-SOD, U/mg prot). **D** The content of malondialdehyde (MDA, nmol/mg prot). Values are mean ± SD, *n* = 3. The significant differences among groups are represented by different lowercase letters on the column (*P* < 0.05). NC: the basic diet; HC: CPC substitutes 75% of the FM protein in the NC diet; HCH: adding 1.25 g/kg of HT to the HC diet

In comparison to the NC and HCH groups, fish on the administration of the HC diet resulted in a reduction of the level of T-AOC and the activities of T-GSH and T-SOD in the fish intestine, while concurrently elevating the content of MDA (Fig. 4A–D; *P* < 0.05).

### Serum immunity

**Fig. 5:**
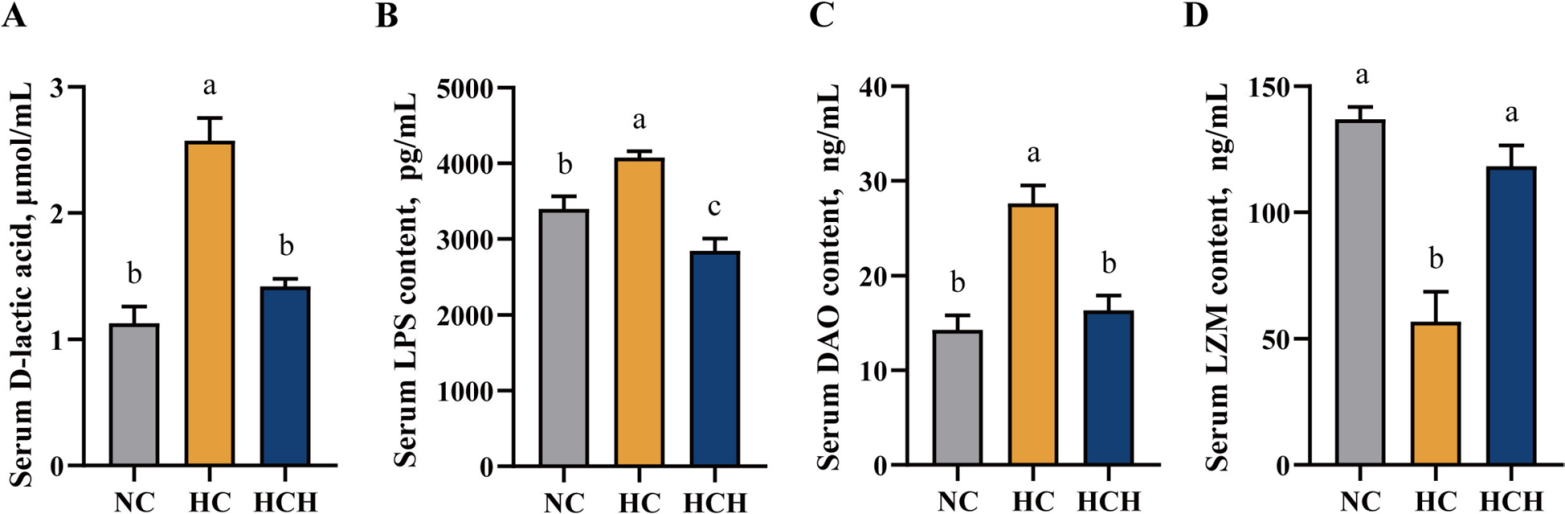
Effect of HT supplementation in the high CPC diet on the serum immunity. **A** The concentration of D-lactic acid, μmol/mL. **B** The concentration of lipopolysaccharides (LPS, pg/mL). **C** The concentration of diamine oxidase (DAO, ng/mL). **D** The concentration of lysozyme (LZM, ng/mL). Values are mean ± SD, *n* = 3. The significant differences among groups are represented by different lowercase letters on the column (*P* < 0.05). NC: the basic diet; HC: CPC substitutes 75% of the FM protein in the NC diet; HCH: adding 1.25 g/kg of HT to the HC diet

The administration of the HC diet caused a marked elevation in the serum concentrations of D-lactic acid, LPS and DAO in fish than that under the NC and HCH treatments, while the concentration of LZM in the serum showed the opposite trend (Fig. 5A–D; *P* < 0.05).

### Intestinal inflammation

**Fig. 6:**
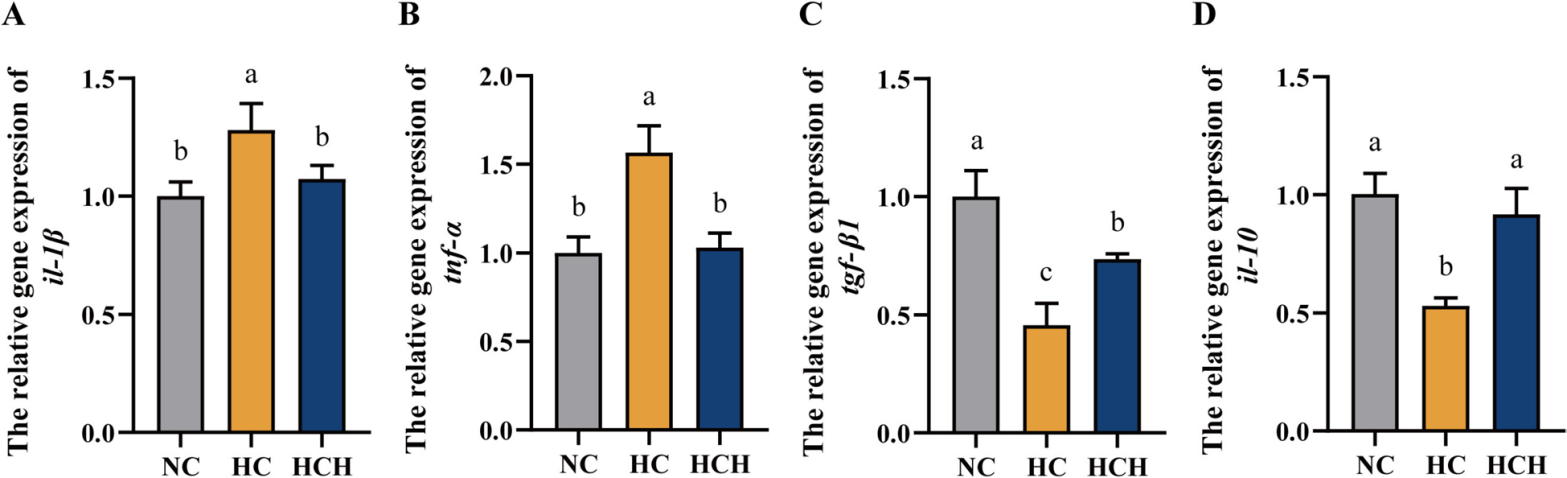
Effect of HT supplementation in the high CPC diet on intestinal inflammation. **A** The relative gene expression of interleukin-1β (*il-1β*). **B** The relative gene expression of tumor necrosis factor-α (*tnf-α*). **C** The relative gene expression of transforming growth factor-β1 (*tgf-β1*). **D** The relative gene expression of interleukin-10 (*il-10*). Values are mean ± SD, *n* = 3. The significant differences among groups are represented by different lowercase letters on the column (*P* < 0.05). NC: the basic diet; HC: CPC substitutes 75% of the FM protein in the NC diet; HCH: adding 1.25 g/kg of HT to the HC diet

Fish under HC treatment had higher intestinal expression levels of interleukin-1β (*il-1β*) and *tnf-α* relative to those under the NC and HCH treatments (Fig. 6A and B; *P* < 0.05), while the expression levels of anti-inflammatory factors (transforming growth factor-β1 (*tgf-β1*) and interleukin-10 (*il-10*)) were notably downregulated (Fig. 6C and D; *P* < 0.05).

### Intestinal microbiota

**Fig. 7:**
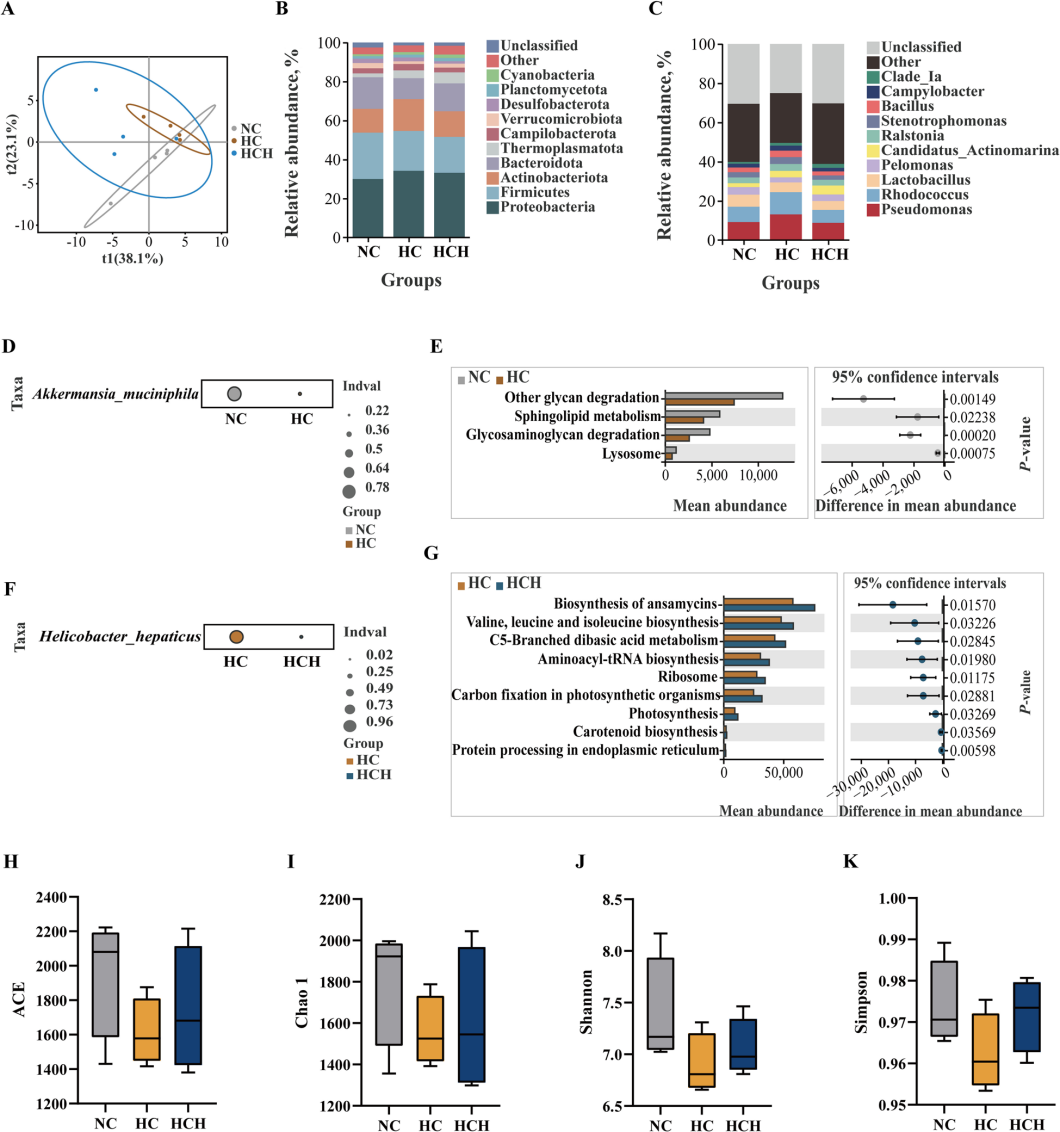
Effect of HT supplementation in the high CPC diet on intestinal microbe. **A** Partial least squares-discriminate analysis (PLS-DA). **B** Major species composition at the phylum level. **C** Major species composition at the genus level. **D** Analysis of indicator species at the species level (NC-vs-HC). **E** Functional prediction analysis of microorganisms (NC-vs-HC). **F** Analysis of indicator species at the species level (HC-vs-HCH). **G** Functional prediction analysis of microorganisms (HC-vs-HCH). **H** ACE index. **I** Chao 1 index. **J** Shannon index. **K** Simpson index. Values are mean ± SD, *n* = 4. The significant differences among groups are represented by different lowercase letters on the column (*P* < 0.05). NC: the basic diet; HC: CPC substitutes 75% of the FM protein in the NC diet; HCH: adding 1.25 g/kg of HT to the HC diet

Variations in the composition of the intestinal microbiota were observed as a result of different dietary treatments (Fig. 7A). The dominant phyla of intestinal microorganisms in fish across the three dietary treatments were Proteobacteria, Firmicutes, Actinobacteriota, Bacteroidota, and Thermoplasmatota (Fig. 7B). Compared to fish under the NC and HCH treatments, the relative abundance of Bacteroidota in the intestine of fish under the HC treatment was reduced, while the levels Proteobacteria and Actinobacteriota were increased (Fig. 7B). The dominant genera of intestinal microorganisms in fish across the three dietary treatments were *Pseudomonas*, *Rhodococcus*, *Lactobacillus*, *Pelomonas*, and *Candidatus_Actinomarina* (Fig. 7C). In comparison with fish under the NC and HCH treatments, fish under the HC treatment exhibited an increased relative abundance of *Rhodococcus* and *Pseudomonas*. The relative abundance of *Lactobacillus* and *Pelomonas* was reduced in fish under the HC treatment relative to those under the NC treatment (Fig. 7C). Indicator species analysis at the species level showed that the fish intestine in the NC treatment had a higher abundance of *Akkermansia_muciniphila* than those in the HC treatment (Fig. 7D); the fish intestine in the HC treatment exhibited a higher abundance of *Helicobacter_hepaticus* than those in the HCH treatment (Fig. 7F). In terms of the predicted microbial community function using PICRUSt, the HC treatment resulted in lower functional involvement by intestinal microorganisms in fish compared to the NC and HCH treatments (Fig. 7E and G). The diversity indices (ACE, Chao1, Shannon, and Simpson) of intestinal microorganisms in fish did not change markedly with the alteration of diets (Fig. 7H–K; *P* > 0.05).

### Intestinal metabolite analysis

**Fig. 8:**
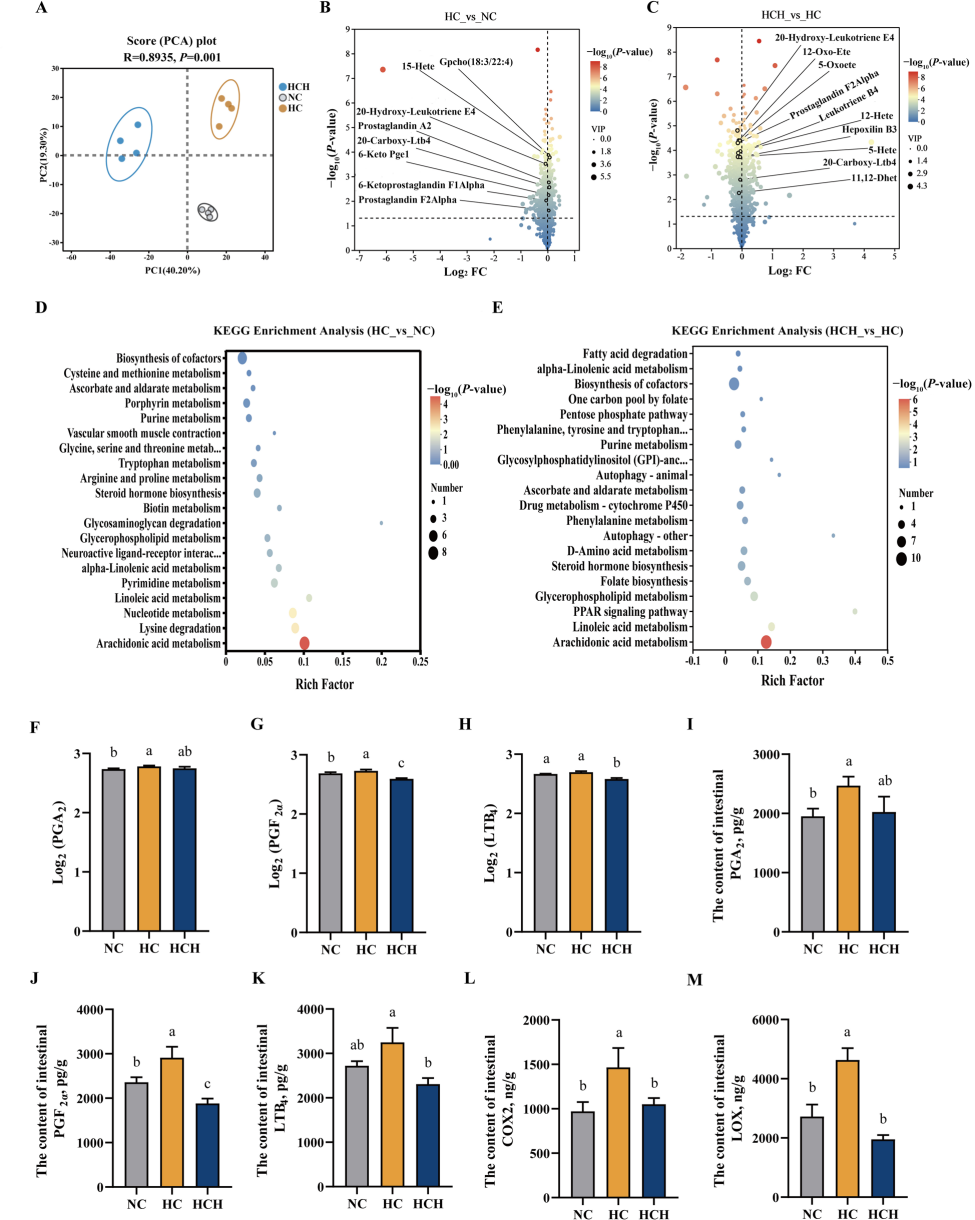
Analysis of intestinal metabolite. **A** Principal component analysis (PCA). **B** Volcano plot (HC group vs. NC group). **C** Volcano plot (HCH group vs. HC group). **D** The top 20 KEGG metabolic pathways with significant differences between HC group and NC group. **E** The top 20 KEGG metabolic pathways with significant differences between HCH group and HC group. **F** The abundance of the differential metabolite: Prostaglandin A_2_ (PGA_2_). **G** The abundance of the differential metabolite: Prostaglandin F_2α_ (PGF_2α_). **H** The abundance of the differential metabolite: Leukotriene B_4_ (LTB_4_). **I** The content of intestinal PGA_2_. **J** The content of intestinal PGF_2α_. **K** The content of intestinal LTB_4_. **L** The content of intestinal cyclooxygenase 2 (COX2). **M** The content of intestinal lipoxygenases (LOX). Values are mean ± SD, *n* = 4 for the analysis of metabolome, *n* = 3 for the detection of the content of PGA_2_, PGF_2α_, LTB_4_, COX2, and LOX. The significant differences among groups are represented by different lowercase letters on the column (*P* < 0.05). NC: the basic diet; HC: CPC substitutes 75% of the FM protein in the NC diet; HCH: adding 1.25 g/kg of HT to the HC diet

There was no overlap among the samples of different groups (Fig. 8A). There were 755 differential metabolites between the HC and NC groups, of which 455 were upregulated and 300 were downregulated in the HC group (Fig. 8B; *P* < 0.05). Relative to the HC group, the HCH group had 256 upregulated metabolites and 591 downregulated metabolites (Fig. 8C; *P* < 0.05). Among the two comparative groups (HC vs. NC, HCH vs. HC), the KEGG pathway with the most significant differences was arachidonic acid metabolism (Fig. 8D and E; *P* < 0.05). In the arachidonic acid metabolism pathway, the HC group had 6 upregulated differential metabolites and 2 downregulated differential metabolites compared with the NC group (Fig. 8B; *P* < 0.05). The HCH group had 10 differential metabolites compared with the HC group, and all of that were downregulated (Fig. 8C; *P* < 0.05). The typical arachidonic acid metabolite PGA_2_ was present in higher abundance and content in the HC group than in the NC group (Fig. 8F and I; *P* < 0.05). The abundance and content of PGF_2α_ in the HC group were significantly elevated than those in the NC and HCH groups (Fig. 8G and J; *P* < 0.05). Furthermore, the abundance and content of LTB_4_ were significantly reduced in the HCH group than in the HC group (Fig. 8H and K; *P* < 0.05). The contents of the key enzymes in arachidonic acid metabolism, COX2 and LOX, were significantly elevated in the HC group than in the other two groups (Fig. 8L and M; *P* < 0.05).

### Intestinal transcriptome

**Fig. 9:**
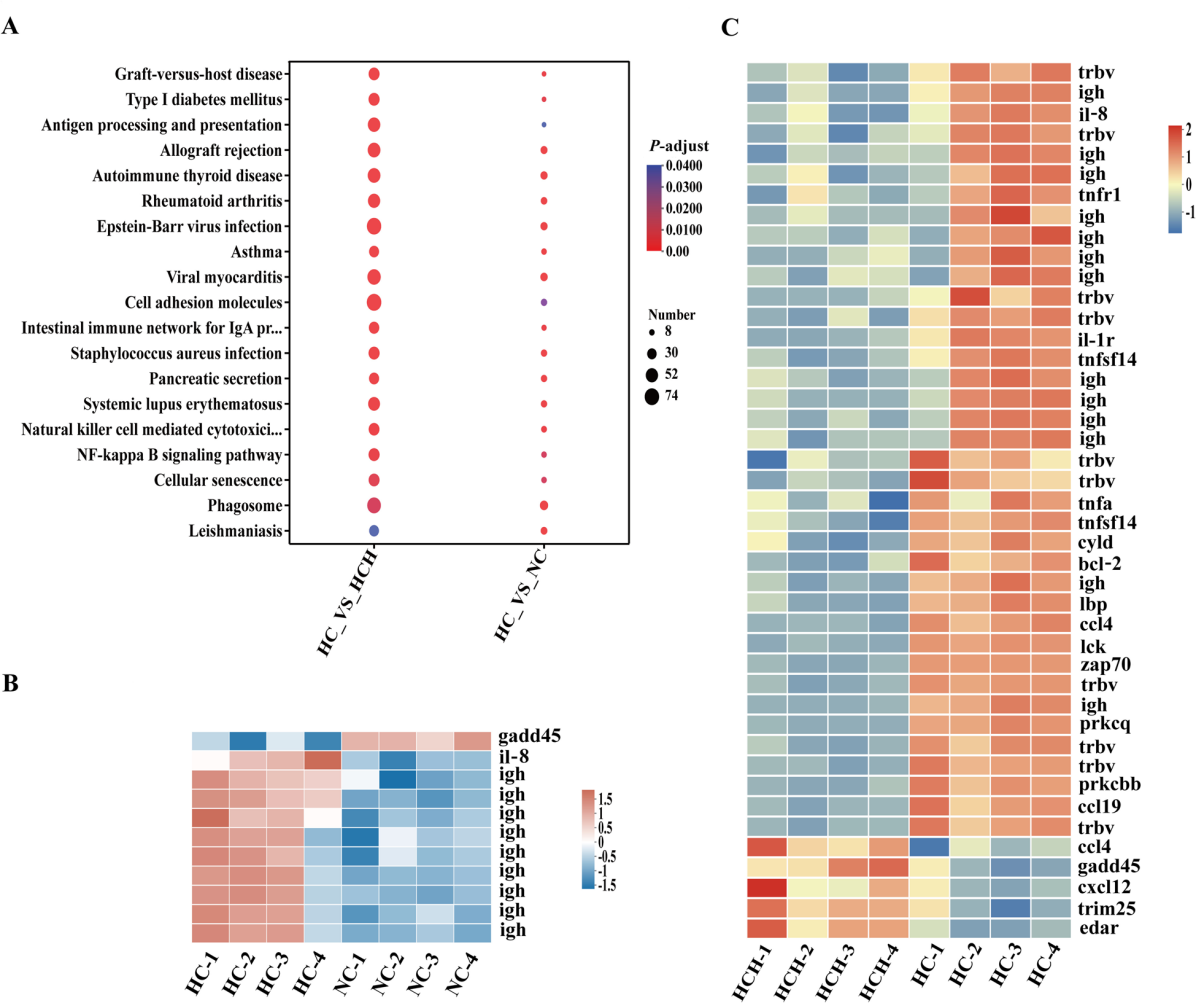
Analysis of intestinal transcriptome. **A** The top 20 pathways with the highest enrichment levels that intersect between the two comparison groups (HC group vs. NC group; HC group vs. HCH group). **B** Genes with significant differences in the NF-κB signaling pathway between the NC group and the HC group. **C** Genes with significant differences in the NF-κB signaling pathway between the HCH group and the HC group. *n* = 4. *P*-adjust < 0.05. Fold change: 2. NC: the basic diet; HC: CPC substitutes 75% of the FM protein in the NC diet; HCH: adding 1.25 g/kg of HT to the HC diet

To enhance the pertinence of comparisons, we adopted pairwise comparisons to analyze the results of transcriptome. The samples from the HC group were distinctly separated from those of the HCH group with no overlap, whereas the samples from the HC group had overlap with the NC group (Fig. S2). The top 20 overlapping KEGG pathways between the two comparative groups (HC vs. NC, HC vs. HCH) are showed in Fig. 9A, among which the NF-κB signaling pathway is directly related to arachidonic acid metabolism. Between the HC and NC groups, within the NF-κB signaling pathway, growth arrest and DNA damage-inducible protein (*gadd45*) was upregulated in the NC group, while the genes interleukin-8 (*il-8*) and immunoglobulin heavy chain (*igh*) showed the opposite trend (Fig. 9B; *P* < 0.05). Between the HC and HCH groups, within the NF-κB signaling pathway, apart from C–C motif chemokine 4 (*ccl4*), *gadd45*, stromal cell-derived factor 1 (*cxcl12*), E3 ubiquitin/ISG15 ligase TRIM25 (*trim25*), and ectodysplasin A receptor (*edar*), all other significantly differentially expressed genes were upregulated in the HC group (Fig. 9C; *P* < 0.05).

### Correlation analysis

**Fig. 10:**
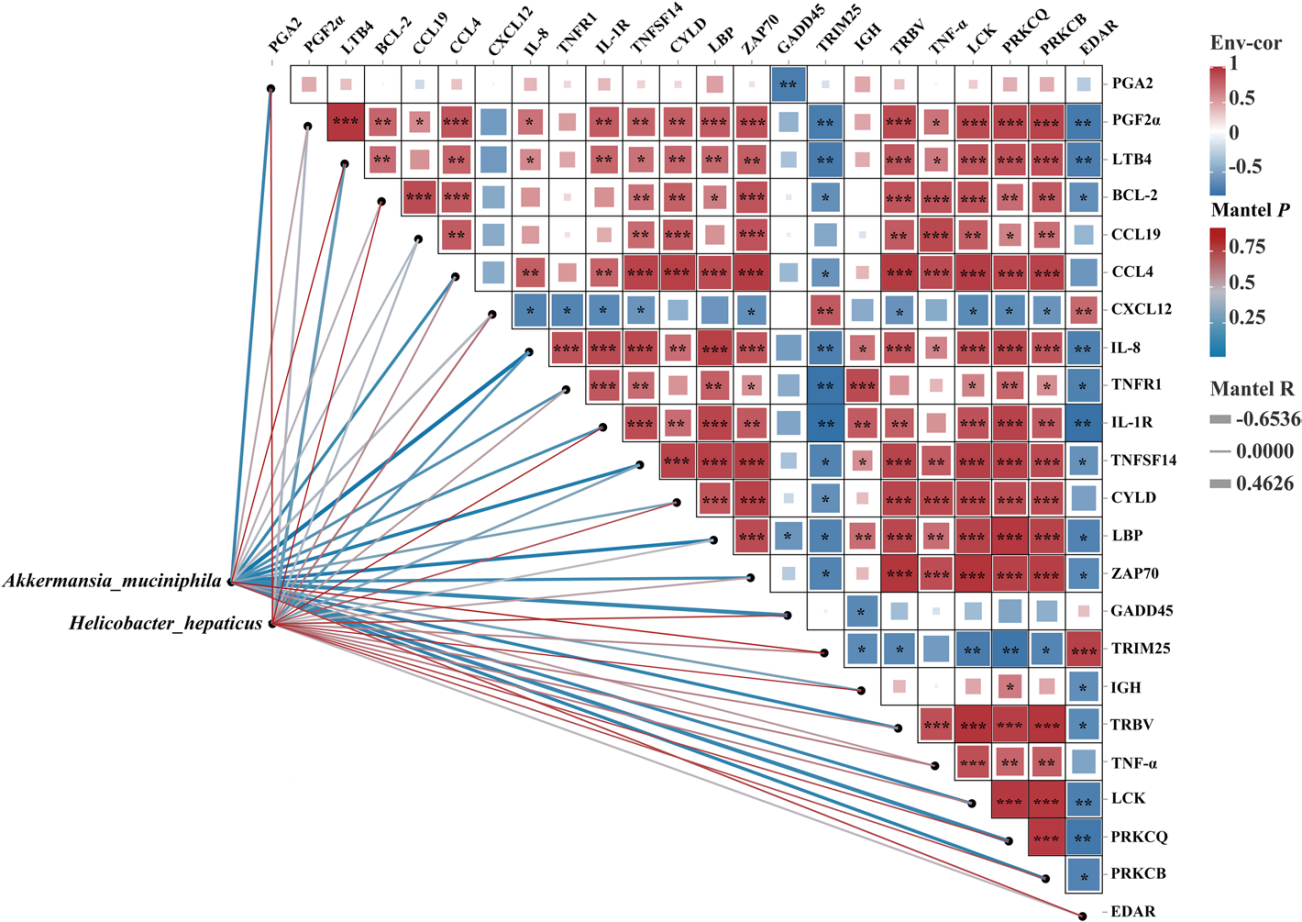
Correlation analysis. Indicator species at the microbial species level were used for correlation analysis with the typical metabolites of arachidonic acid and the significantly differentially expressed genes in the NF-κB signaling pathway. ^*^P < 0.05, ^**^P < 0.01, ^***^P < 0.001

The results of the correlation analysis are showed in Fig. 10. Regarding the correlation analysis between microbiota and metabolites, *Akkermansia_muciniphila* and *Helicobacter_hepaticus* both showed no significant correlation with the arachidonic acid metabolites (PGA_2_, PGF_2α_, and LTB_4_) (*P* > 0.05). Regarding the correlation analysis between microbiota and transcriptome, *Akkermansia_muciniphila* had a significant negative correlation with *il-8* (*P* < 0.05). *Helicobacter_hepaticus* showed no significant correlation with the significantly differentially expressed genes within the NF-κB signaling pathway (*P* > 0.05). Regarding the correlation analysis between the transcriptome and metabolome, PGA_2_ had a significant negative correlation with *gadd45* (*P* < 0.05). PGF_2α_ had significant positive correlations with protein kinase C, beta (*prkcb*), protein kinase C, theta (*prkcq*), LCK proto-oncogene, Src family tyrosine kinase (*lck*), *tnf-a*, T-cell receptor beta chain V region (*trbv*), tyrosine-protein kinase ZAP-70 (*zap70*), BPI fold containing family C (*lbp*), ubiquitin carboxyl-terminal hydrolase CYLD (*cyld*), tumor necrosis factor ligand superfamily member 14 (*tnfsf14*), interleukin-1 receptor type 1 (*il-1r)*, *il-8*, *ccl4*, chemokine (C–C motif) ligand 19 (*ccl19*), and *bcl-2*, while it was notable negative correlations with *edar* and *trim25* (*P* < 0.05). LTB_4_ had significant positive correlations with *prkcb*, *prkcq*, *lck*, *tnf-a*, *trbv*, *zap70*, *lbp*, *cyld*, *tnfsf14*, *il-1r*, *il-8*, *ccl4*, and *bcl-2*, while it showed significant negative correlations with *trim25* and *edar* (*P* < 0.05).

## Discussion

To more effectively address the scarcity resources and the elevated costs associated with FM, the utilization of CPC as an alternative in aquaculture feeds has become increasingly prevalent [4, 6, 19]. Nonetheless, it is important to note that excessively high proportion of CPC substitution for FM may adversely influence fish growth [6, 7, 35]. The findings of the present study aligned with the prior research, indicating that the HC diet treatment inhibited the growth of largemouth bass. Although the study on grass carp revealed that the dietary HT could improve feed intake [24], feed intake was not enhanced by the addition of the HT in this research, which is consistent with our previous results on condensed tannin [31], and this may be due to the astringent taste of tannins and the different feeding habits of fish, which lead to the differences in palatability [20]. However, the HCH diet was showed to effectively enhance the weight gain rate of fish in this study. As is consistent with our study, the benefits of the dietary HT on fish growth have been studied, as evidenced by the promotion of weight gain in pearl gentian grouper [36], Pacific white shrimp (*Litopenaeus vannamei)* [37], and largemouth bass [33]. Nevertheless, a study has indicated that the dietary HT inhibited the growth of largemouth bass fed with the high-carbohydrate diet (additive amount of HT: 0.2 g/kg) [38]. The conflicting results may be attributed to the growth-inhibitory impact of high-carbohydrate diets on largemouth bass [39, 40].

The ability of tannins to bind with proteins has led to their classification as anti-nutritional factors in plant-derived proteins [20]. According to the different chemical structures of tannins, tannins can be divided into three categories, among which condensed tannins are polymers constituted by the linkage of flavan-3-ol units, including catechin and epicatechin, through carbon–carbon bonds [41]. Additionally, due to their substantial molecular weights, the polymerized structures of condensed tannins facilitate interactions with biological macromolecules such as proteins and polysaccharides [20]. Therefore, excessive intake of condensed tannins by fish may lead to the formation of stable complexes with proteins, resulting in a decrease in the activity of digestive enzymes in largemouth bass and Japanese seabass (*Lateolabrax japonicus*) [20, 32, 42]. HTs are formed by the combination of a polyol (usually glucose) core and phenolic acids (such as gallic acid or ellagic acid) through ester bonds [41]. The phenolic acid groups in their structures are abundant in hydroxyl groups, which can provide hydrogen atoms, thus effectively neutralizing free radicals and exhibiting significant antioxidant capacity [43]. Additionally, due to their relatively low molecular weights and the absence of long-chain polymerized structures that are characteristic of condensed tannins, their binding ability to proteins is comparatively weak [20, 43]. HTs mainly interact weakly with the amino acid residues of proteins through the phenolic acids produced by hydrolysis in a non-covalent manner. This interaction is relatively unstable and exerts minimal influence on the structural and functional integrity of proteins [43]. In addition, supplementation with an appropriate amount of the dietary HT has been documented to enhance the activity of intestinal digestive enzymes in grass carp (additive amount: 1.25 g/kg) [24] and obscure puffer (*Takifugu fasciatus*) (additive amount: 2.5 g/kg) [44]. The present result is consistent with these findings. However, a study on Chinese seabass revealed that the addition of 2 g/kg HT to the basal diet led to a decrease in the activity of intestinal digestive enzymes, with the negative effects of condensed tannin (2 g/kg) being even more pronounced [45]. Differences in fish species and the dosage of the dietary HT may affect its influence on intestinal digestive enzymes in fish. On the other hand, the upregulation of gene expression of intestinal *muc-2* in the HCH group were conducive to the secretion of various digestive enzymes [46]. Additionally, tissue damage, structural alterations, and oxidative stress can collectively impair the functionality of intestinal epithelial cells, resulting in decreased enzyme secretion and consequently affecting food digestion and nutrient absorption [46]. Therefore, the maintenance of intestinal structural integrity and the enhancement of antioxidant capacity by the dietary HT may contribute to the secretion of digestive enzymes in fish.

The preservation of intestinal integrity is essential for sustaining intestinal barrier function, immune response, nutrient absorption, regulation of inflammation, and microbial homeostasis, all of which collectively influence overall health status [12, 47]. According to previous research, the dietary HT preserved the structural integrity of intestine in Chinese seabass [48] and facilitated the development of intestinal villi in pearl gentian grouper [36] and zebrafish [49]. Similarly, in this study, the HC treatment induced damage to intestinal tissue in largemouth bass, while the HCH treatment preserved the integrity of the intestinal villi, with fewer apoptotic cells in intestinal villi, and an upregulation in tight junction protein genes and anti-apoptotic genes expression. Which is consistent with the research findings on condensed tannin [31]. Furthermore, the beneficial impact of tannins on intestinal tight junction proteins has been substantiated through studies conducted on Chinese seabass [50, 51]. Which could reduce intestinal permeability, thereby limiting the translocation of D-lactic acid, LPS and DAO into the bloodstream through the intestinal barrier, ultimately mitigating the initiation of systemic inflammation [46]. This supports our findings, as the addition of the dietary HT reduced the concentrations of serum D-lactic acid, LPS, and DAO in largemouth bass.

The dietary composition exerts a considerable influence on the composition and functionality of the intestinal microbiota, while the intestinal microbiota, in turn, can influence the digestion, utilization of the diet, as well as intestinal health [7, 17, 46]. The abundance of potential pathogens such as *Pseudomonas* [52], *Rhodococcus* [53], *Ralstonia* [54], and *Stenotrophomonas* [55] was observed to be highest in the intestines of fish under the HC treatment, while the abundance of the beneficial bacterium *Lactobacillus* [56] was found to be lowest in this group, compared to the NC treatment. In addition, the probiotic *Akkermansia_muciniphila* [46] in the NC group was significantly more abundant than that in the HC group. While the pathogenic bacteria *Helicobacter_hepaticus* [57] in the HC group was significantly more abundant than that in the HCH group. Which suggests that the dietary HT may potentially optimize intestinal microbiota.

HT has been found to be utilized by intestinal microorganisms to produce some beneficial metabolites, especially small-molecule phenolic compounds such as gallic acid and ellagic acid, which have anti-inflammatory and antioxidant effects [43, 58]. Metabolomic analysis identified arachidonic acid metabolism, which is the primary network for production of inflammatory mediators and the induction of inflammation [59], as the most significantly different metabolic pathway between the two comparison groups (NC vs. HC, HC vs. HCH). Cyclooxygenase (COX) and LOX are two key enzymes involved in arachidonic acid metabolism. Free arachidonic acid is further metabolized into various bioactive mediators by COX, LOX, and cytochrome P450 [26]. The COX enzyme exists in two isoforms, COX1 and COX2, with COX2 being either minimally or not expressed under normal physiological conditions but markedly upregulated in diverse inflammatory and carcinogenic environments [26]. Moreover, PGF_2α_ and PGA_2_, which are typical products of the COX metabolic pathway, exert adverse effects on the inflammatory response. The LTB_4_ is a pro-inflammatory mediator generated by the LOX metabolic pathway [27]. The research found that the levels of these key enzymes and metabolites with pro-inflammatory properties were notably higher in the HC group than in the HCH group, suggesting that the dietary HT may has a positive effect on the occurrence or progression of inflammation in the fish intestine. Additionally, this study indicated that the HCH treatment downregulated pro-inflammatory factors while upregulated anti-inflammatory factors in the intestine, further supporting the aforementioned results and corroborating previous research outcomes [2, 35]. Similarly, the dietary HT has been observed to downregulate the expression levels of pro-inflammatory factors such as *cox2*, *il-1β*, and *tnf-α* in the intestine of zebrafish, while upregulating the expression of the anti-inflammatory factor *il-10* [18, 49]. Additionally, the dietary HT enhanced the expression of lysozyme and antimicrobial peptides in whiteleg shrimp infected with *V. alginolyticus* [23].

The transcriptome results showed that among the top 20 significant KEGG pathways with intersections between the two comparison groups in the present study, the pathway directly related to arachidonic acid metabolism was the NF-κB signaling pathway [28]. Within the NF-κB signaling pathway, both T-cell receptor (TCR) and B-cell receptor (BCR) pathways, which belong to adaptive immunity, can activate the NF-κB signaling pathway [60]. The BCR pathway plays a crucial role in the immune response mediated by B cells [60]. The *igh*, has multiple variant forms, allowing B cells to recognize different antigens and activate signaling pathways [61]. The TCR pathway constitutes a critical signaling cascade in the modulation of the T cell immune response [62]. The *trbv* encodes different variable forms of the beta chain, assisting T-cells in recognizing a wide range of antigens, which subsequently activates the TCR signaling pathway [62]. Activation of TCR signaling pathway leads to the mobilization of intracellular calcium ions and the activation of NF-κB signaling. Similarly, activated *prkcb* in the BCR pathway, or *prkcq* in TCR pathway can recruit the CARMA1/Bcl-10/MALT1 complex, thereby activating NF-κB signaling [29]. In present research, compared to fish under the HC treatment, the NC diet resulted in the downregulation of intestinal gene expression of *igh*, while the HCH diet led to the downregulation of gene expression for *igh*, *trbv*, *prkcb*, and *prkcq*, suggesting that the intake of high doses of CPC may induce intestinal adaptive immunity in largemouth bass. Activated NF-κB signaling releases transcription factors to translocate into the nucleus, regulating the transcription of a series of genes involved in immune response, cell survival, proliferation, and inflammatory reactions, such as *il-8* and *gadd45* [60]. As showed in our study, the expression level of the *il-8* was significantly lower in the NC and HCH groups compared to the HC group, while the expression of *gadd45* was the opposite. The *il-8* mainly plays a promoting role in the development of inflammation, contributing to the recruitment of immune cells to the site of inflammation and the enhancement of the inflammatory response. In contrast, the upregulation of *gadd45* may be a form of cellular self-limitation mechanism, which helps to prevent the excessive activation of inflammation [29]. Which suggests that the dietary HT may have a positive regulatory effect on the development of intestinal inflammation.

In addition, upon activation, NF-κB translocates to the nucleus and promotes the expression of *cox2*, which involves in the synthesis of prostaglandins, thereby directly driving the generation of pro-inflammatory mediators such as PGF_2α_ and PGA_2_ [28]. Which can also upregulate 5-lipoxygenase, facilitating the synthesis of leukotrienes, such as LTB₄ [63]. Meanwhile, the metabolites of arachidonic acid can regulate the activity of NF-κB through feedback mechanisms, leading to its sustained activation and the formation of an inflammatory amplification loop [28]. Through correlation analysis, the present research identified significant associations between the metabolites of arachidonic acid, specifically PGA_2_, PGF_2α_, and LTB_4_, and the pertinent genes within the NF-κB signaling pathway. Specifically, PGA_2_ had a significant negative correlation with *gadd45*, while PGF_2α_ and LTB_4_ exhibited significant positive correlations with *il8*, *trbv*, *prkcb*, and *prkcq*. This suggests that the dietary HT may alleviate intestinal inflammation in largemouth bass by positively regulating the metabolism of arachidonic acid and the NF-κB signaling pathway.

## Conclusion

It was concluded that supplementing the diet with the HT (1.25 g/kg) mitigated the adverse effects induced by the high level of CPC substitution, resulting in an increase in weight gain rate, an improved feed conversion ratio, preservation of the integrity of intestinal structure, enhanced activity of intestinal digestive enzymes and antioxidant system, the optimization of intestinal microbiota, and a downregulation in the gene expression of pro-inflammatory factors. Additionally, transcriptomic and metabolomic results demonstrated that the dietary HT may alleviate intestinal inflammation by playing a positive role in regulating NF-κB signaling pathway and arachidonic acid metabolism.

## Supplementary Information

Additional file 1: Table S1 Sequences of primer used in qPCR. Fig. S1 Metabolomic analysis of two types of tannins. Fig. S2 Principal component analysis (PCA) of three groups.

## Data Availability

Data will be made available on request.

## Abbreviations

*bcl-2*: B-cell lymphoma-2
*bcl-xl*: B-cell lymphoma-extra large
CPC: Cottonseed protein concentrate
CT: Condensed tannin
CF: Condition factor
COX2: Cyclooxygenase 2
COX: Cyclooxygenase
*ccl4*: C–C motif chemokine 4
*cxcl12*: Stromal cell-derived factor 1
*ccl19*: Chemokine (C–C motif) ligand 19
*cyld*: Ubiquitin carboxyl-terminal hydrolase CYLD
DAO: Diamine oxidase
*edar*: Ectodysplasin A receptor
FM: Fishmeal
FCR: Feed conversion ratio
FI: Feed intake
FBW: Final body weight
FFN: Final fish number
FBL: Final body length
*gadd45*: Growth arrest and DNA damage-inducible protein
HT: Hydrolysable tannin
IBW: Initial body weight
IFN: Initial fish number
*il-1β*: Interleukin-1β
*il-10*: Interleukin-10
*il-8*: Interleukin-8
*igh*: Immunoglobulin heavy chain
*il-1r*: Interleukin-1 receptor type 1
LZM: Lysozyme
LPS: Lipopolysaccharide
LTB_4_: Leukotriene B_4_
LOX: Lipoxygenases
*lck*: LCK proto-oncogene, Src family tyrosine kinase
*lbp*: BPI fold containing family C
MDA: Malondialdehyde
PGA_2_: Prostaglandin A_2_
PGF_2α_: Prostaglandin F_2α_
*prkcb*: Protein kinase C, beta
*prkcq*: Protein kinase C, theta
SR: Survival rate
T-AOC: Total antioxidant capacity
T-GSH: Total glutathione
T-SOD: Total superoxide dismutase
*tgf-β1*: Transforming growth factor-β1
*tnf-α*: Tumor necrosis factor-α
*tor*: Target of rapamycin
*trim25*: E3 ubiquitin/ISG15 ligase TRIM25
*trbv*: T-cell receptor beta chain V region
*tnfsf14*: Tumor necrosis factor ligand superfamily member 14
WGR: Weight gain rate
*zo-1*: Zonula occludens-1
*zap70*: Tyrosine-protein kinase ZAP-70
*β-actin*: Beta-actin
