# Supplementary material for: Dietary hydrolysable tannin improves intestinal health of largemouth bass (Micropterus salmoides): insights from NF-κB signaling pathway and arachidonic acid metabolism
Source: J Anim Sci Biotechnol. 2025 Oct 23;16:136. doi: 10.1186/s40104-025-01267-w (PMC12548211; doi:10.1186/s40104-025-01267-w)
Supplement: Supplementary file 1 — Additional file 1: Table S1 Sequences of primer used in qPCR. Fig. S1 Metabolomic analysis of two types of tannins. Fig. S2 Principal component analysis (PCA) of three groups. [file 40104_2025_1267_MOESM1_ESM.docx]

Table S1 Sequences of primer used in qPCR.

| Gene | Primers | Sequence 5′−3′ | GenBank No | Reference |
| --- | --- | --- | --- | --- |
| *β-actin* | F | AAAGGGAAATCGTGCGTGAC | XM_038695351.1 | [35] |
|  | R | AAGGAAGGCTGGAAGAGGG |  |  |
| *zo-1* | F | ATCTCAGCAGGGATTCGACG | XM_038701018.1 | [10] |
|  | R | CTTTTGCGGTGGCGTTGG |  |  |
| *occludin* | F | GATATGGTGGCAGCTACGGT | XM_038715418.1 | [10] |
|  | R | TCCTACTGCGGACAGTGTTG |  |  |
| *claudin-4* | F | TAATCGCTATGGTGGGAGCC | XM_038694323.1 | [10] |
|  | R | GCCCCGATCTCCATCTTCTG |  |  |
| *claudin-1* | F | CCAGGGAAGGGGAGCAATG | XM_038718401.1 | [10] |
|  | R | GCTCTTTGAACCAGTGCGAC |  |  |
| *muc-2* | F | CAGTGCAAGGCAAGTTTGGT | XM_038706087.1 | / |
|  | R | GACTTCCATTCGCCCCAGTT |  |  |
| *tgf-β1* | F | GCTCAAAGAGAGCGAGGATG | XM_038693206.1 | [35] |
|  | R | TCCTCTACCATTCGCAATCC |  |  |
| *tnf-α* | F | CTTCGTCTACAGCCAGGCATCG | XM_038723994.1 | [35] |
|  | R | TTTGGCACACCGACCTCACC |  |  |
| *il-1β* | F | CGTGACTGACAGCAAAAAGAG | XM_038733429.1 | [35] |
|  | R | GATGCCCAGAGCCACAGTTC |  |  |
| *Il-10* | F | CGGCACAGAAATCCCAGAGC | XM_038696252.1 | [35] |
|  | R | CAGCAGGCTCACAAAATAAACATCT |  |  |
| *bcl-xl* | F | CATCCTCCTTGGCTCTGG | XM_038723144.1 | [10] |
|  | R | GGGTCTGTTTGCCTTTGG |  |  |
| *caspase-3* | F | GCTTCATTCGTCTGTGTTC | XM_038699323.1 | [10] |
|  | R | CGAAAAAGTGATGTGAGGTA |  |  |
| *caspase-8* | F | GAGACAGACAGCAGACAACCA | XM_038718636.1 | [22] |
|  | R | TTCCATTTCAGCAAACACATC |  |  |
| *bcl-2* | F | CGCCATCCACAGAGTCCT | XM_038711485.1 | [22] |
|  | R | CCGGAACAGTTCGTCTATCACC |  |  |

Note: *β*-actin: beta-actin; *zo-1*: zonula occludens-1; *occludin*: occludin; *claudin-1/4*: claudin-1/4; *muc-2*: mucin-2; *tgf-β1*: transforming growth factor-β1; *tnf-α*: tumor necrosis factor-α; *il-1β*: interleukin-1β; *il-10*: interleukin-10; *bcl-xl*: B-cell lymphoma-extra large; *caspase3/8*: caspase3/8; *bcl-2*: B-cell lymphoma-2.


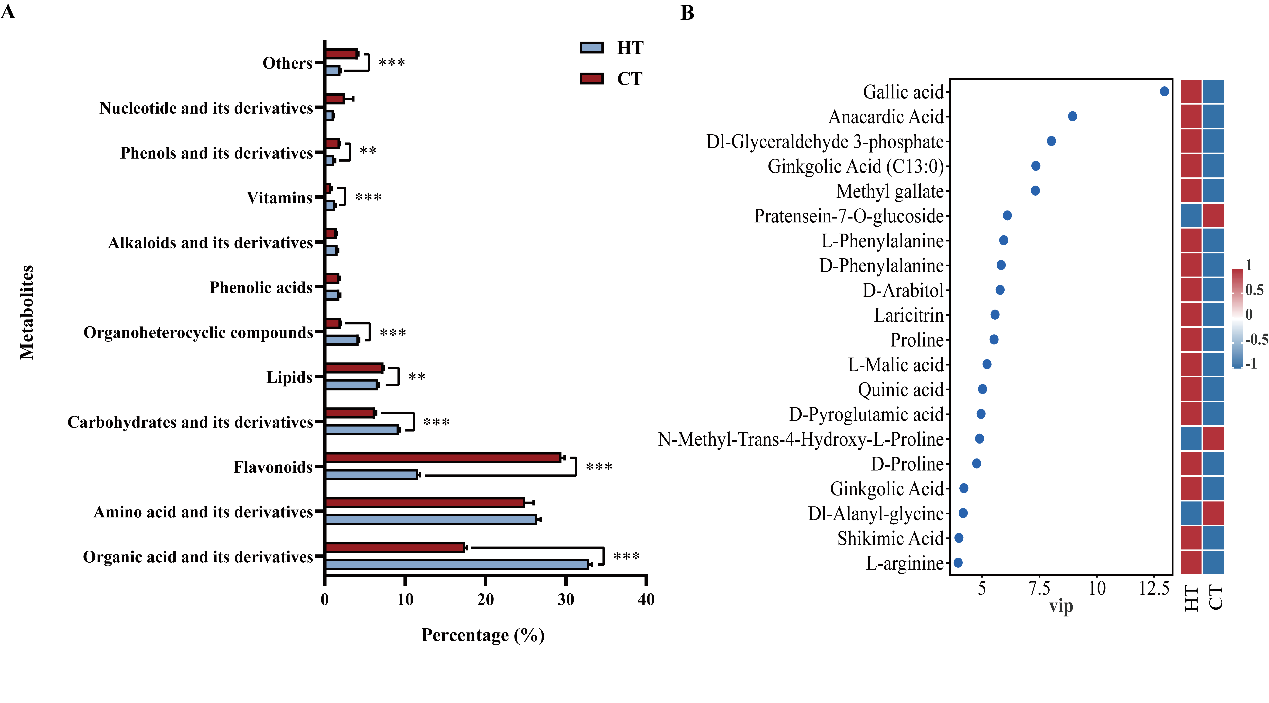


**Fig. S1** Metabolomic analysis of two types of tannins. **A** Metabolite category. **B** Analyzing differential metabolites between two types of tannins through Variable Importance in Projection (VIP). Substances accounting for less than 1% were classified into “others”. “Others” include amines, phenylpropanoids and polyketides, benzene and substituted derivatives, alcohols and polyols, polyamines, naphthalenes, terpenoids, organosulfur compounds, organooxygen compounds, and phytohormones. n = 3. The significant differences between groups are represented by * on the column (*P* < 0.05), ** means *P* < 0.01, *** means *P* < 0.001. HT: hydrolysable tannin extracted from gallnut; CT: condensed tannin extracted from quebracho bark.


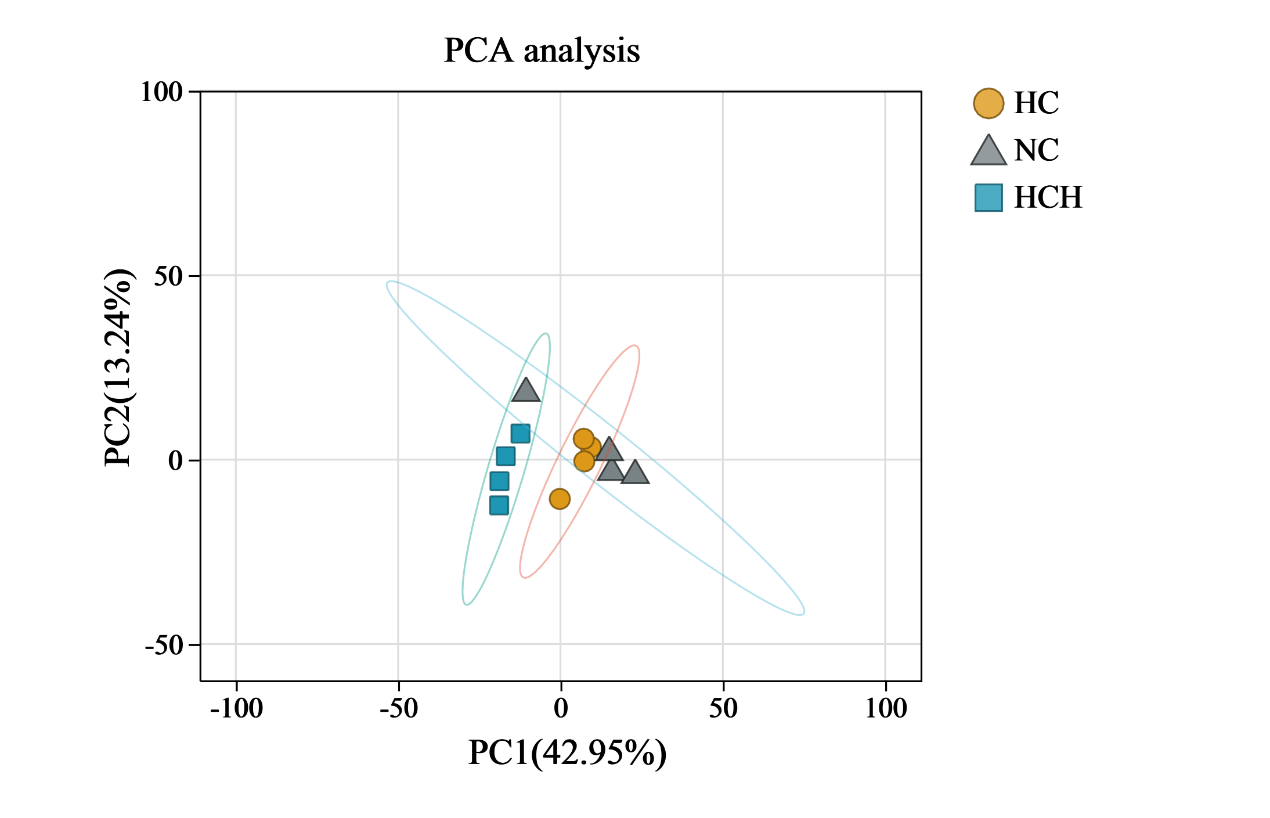


**Fig. S2** Principal component analysis (PCA) of three groups.
